# Immobilized microbial cell biocatalysts: An annotated selection of World Wide Web sites relevant to the topics in *microbial biotechnology*

**DOI:** 10.1111/1751-7915.13787

**Published:** 2021-03-06

**Authors:** Lawrence P. Wackett

**Affiliations:** ^1^ Department of Biochemistry Molecular Biology & Biophysics, BioTechnology Institute University of Minnesota St. Paul MN 55108 USA

## Specialized issue on immobilized biocatalysts

### https://www.mdpi.com/journal/catalysts/special_issues/Immobilized_Biocatalysts

This special issue in the journal *Catalysts* has an extensive collection of articles on the immobilization of enzymes and microbial cells that are expressing key specific enzymes.

## Immobilized whole cell systems

### https://link.springer.com/article/10.1007/s10529‐017‐2300‐y

This review provides a good coverage of microbial cell immobilization methods and provides industrial examples.

## Microbial exoskeleton biocatalyst

### https://www.nature.com/articles/s41598‐019‐40113‐8

This paper describes a layer‐by‐layer encapsulation approach that can be used as part of a whole cell immobilization process.

## Metabolic responses of immobilized microbial cells

### https://www.ncbi.nlm.nih.gov/pmc/articles/PMC6273605/

Microbial cell immobilization can happen naturally, as in a biofilm, or via an engineered process. This paper reviews what is known about the metabolic state of viable immobilized cells.

## One step enzyme expression, cell lysis and immobilization

### https://microbialcellfactories.biomedcentral.com/articles/10.1186/s12934‐015‐0371‐9

This immobilization method is a variation of the whole‐cell theme. The study explored whole cell enzyme expression, along with expression of a phage lytic process that allows enzyme leakage and subsequent immobilization.

## Whole cell biocatalysis

### https://www.creative‐enzymes.com/service/whole‐cell‐biocatalysts_408.html

This commercial website briefly describes the steps in whole cell biocatalyst develop, including surface expression of enzymes.

## Whole cell biocatalysis in non‐conventional media

### https://academic.oup.com/jimb/article/12/2/76/5987798

While many biocatalytic reactions are performed in aqueous media, it is often desirable, or even necessary, to conduct the biotransformation reactions in organic solvents. This article reviews some of the issues involved in whole cell biocatalysis in organic solvents.

## Whole cell Actinobacteria as biocatalysts

### https://www.frontiersin.org/articles/10.3389/fmicb.2019.00077/full

Actinobacteria are responsible for producing most of the known natural products and are important in waste chemical degradation. This paper focuses more on the biocatalysis aspect of the bacteria rather than methodologies.

## Whole cell immobilization

### http://old‐biomikro.vscht.cz/vyuka/ibs/Prednaska6.pdf

This slide set offers a general primer on whole cell immobilization.

## Immobilized cells for winemaking

### https://core.ac.uk/download/pdf/55632376.pdf

Traditionally, wine making has used unattached cells in the juice undergoing fermentation. This study explored the use of immobilized cell processes for winemaking.

## Coculture microbial cell immobilization

### https://pubs.acs.org/doi/10.1021/acs.iecr.0c01867

Most often, microbial cell immobilization is performed with a single pure culture. However, it can be advantageous to combine microbes with different metabolic capabilities and this paper deals with cell immobilization technology to enhance coculture biotransformations.

## Patents on microbial immobilization technology

### http://antaicheng‐group.cn/article‐2008/9.pdf

This review deals with patents on immobilizing materials and use of immobilized cells in bioreactors.

## Technologies for microbial sensors

### https://www.frontiersin.org/articles/10.3389/fbioe.2015.00061/full

This review discusses methods for sensing signals and for fabricating microbial structures and materials for biosensor manufacture.

## Whole cell entrapment techniques

### https://link.springer.com/protocol/10.1007/978‐1‐0716‐0215‐7_25

This web protocol discusses one facet of microbial cell immobilization. In general, cells can be covalently attached to a support, adsorbed, or entrapped within a matrix. This review deals with the latter.

